# Comparison of botulinum toxin (BoNT) injection and lateral internal sphincterotomy (redo-LIS) for recurrent anal fissure treatment

**DOI:** 10.3389/fsurg.2022.988082

**Published:** 2022-09-20

**Authors:** Ahmet Alyanak, Merter Gulen, Bahadır Ege

**Affiliations:** ^1^ Department of General Surgery, Yuksek Ihtisas University Affiliated Medical Park Ankara Private Hospital, Ankara, Turkey; ^2^ Department of General Surgery, Medicana Hospital, Ankara, Turkey

**Keywords:** anal fissure, sphincterotomy, botulinum toxin, recurrent fissure, redo sphincterotomy

## Abstract

**Objective:**

Today's gold standard for treating chronic anal fissure is the Lateral Internal Sphincterotomy (LIS). Botulinum Toxin (BoNT) injection is, on the other hand, an alternative treatment for patients who do not want to have surgical treatment, patients undergoing chemotherapy, patients of high risk for surgery, and those who have the risk of anal incontinence (e.g., elderly, past anorectal surgery, vaginal multiple births, etc.). The aim of this study is to compare the effectiveness of BoNT and redo-LIS for treatment of post-LIS recurrent chronic anal fissure, and reveal differences if any.This study aims to compare redo-LIS and BoNT injection for treating post-LIS recurrent anal fissure.

**Material and method:**

Nineteen patients who received LIS treatment and then redo-LIS or BoNT injection due to recurrence in the follow-up were included in this study. Group I (redo-LIS group) include 11 patients and group 2 (BoNT group) includes 8 patients. Their data on age, sex, anal incontinence scores and pain (VAS score) score as well.

**Results:**

During the 3-month post-surgery follow-up period, there was statistically significant difference (*p* < 0.01) between groups by pain. No deterioration in the incontinence scores of patients in the group during the 6-month post-surgery period.

**Conclusion:**

This study demonstrates that redo lateral internal sphincterotomy (LIS) is a reliable method for patients who received LIS but developed recurrent chronic anal fissure, and achieves successful results in terms of recurrence and relief of pain.

## Introduction

Anal fissure is a frequent disease in the society with a lifetime incidence of 11% (1). For treatment of acute anal fissure, healing is possible by exercising high-fibre diet, taking warm-sitz bath, and applying cremes that reduce internal sphincter pressure (2). Today's gold standard for treating chronic anal fissure is the Lateral Internal Sphincterotomy (LIS) (3). Botulinum Toxin (BoNT) injection is, on the other hand, an alternative treatment for **patients who do not want to have surgical treatment**, patients undergoing chemotherapy, patients of high risk for surgery, and those who have the risk of anal incontinence (e.g., elderly, past anorectal surgery, vaginal multiple births, etc.) (4, 5).

Anal fissure is one of the most frequent benign anorectal diseases (1). The most frequent complaint at clinical visits includes painful defecation accompanied by rectal bleeding (6). Pain particularly may reduce the quality of life of patients (7, 8). Chronic anal fissure is accompanied by hypertrophic papilla along whose edge internal sphincter muscle fibres become visible (9). Physical and chemical agents are used basically to reduce sphincter pressure for treating acute anal fissure. The guidelines of American Society of Colon and Rectal Surgeons (ASCRS) recommend using faeces softeners, high-fibre diet and warm-sitz bath (6). Such treatment methods are currently under discussion (10). Lateral Internal Sphincterotomy (LIS) is indicated as the gold standard for anal fissure treatment (6, 11, 12). The LIS treatment executed by surgeons experienced in proctology proved to achieve better results in terms of removing symptoms and shorter healing time (13).

Recurrence is rare after treatment of chronic anal fissure by LIS. The reason for recurrency, if it ever occurs, is generally the inadequately performed LIS which does not ensure compete healing and causes patients to develop non-healing fissure or early recurrence. The patient continues to experience, though may be at lower intensity, such pre-LIS symptoms as pain, bleeding, avoidance of defecation, anal spasm and pain for 1 to 2 h following defecation.

In case of anal fissure that recurs or continues following an inadequate/inappropriate LIS, surgeons or patients mostly avoid redo-LIS. The reason is that a redo-LIS will likely exacerbate the damage to anal sphincter which in turn creates a heightened risk of anal incontinence. **Some patients avoid surgical treatment because sphincterotomy is influenced by the complication of incontinence**. **This has called for the application of alternative treatments such as Botulinum Toxin (BoNT) injection for recurrent fissures.**

BoNT inhibits the secretion of acetylcholine at the presynaptic terminal of neuromuscular combination. The injection works by inducing temporary paralysis in the muscle (14, 15). The effect of BoNT depends on localization, concentration and volume of the injected solution. The volume and concentration varies proportionally to the size of the muscle being treated (16). The literature includes no report that demonstrates an evidence-based result of effect of BoNT on fibrous tissue. Immunological properties of BoNT may stimulate creation of antibodies, which may in turn increase the likelihood of failure in subsequent treatments. No minimum dosage is yet established that will start creation of antibodies (17).

A meta-analysis on 489 patients by Chen et al. revealed that LIS obtained higher healing rates, and a higher rate of incontinence as well; but found no statistically significant difference between LIS and BoNT for other complications. BoNT cases had higher recurrence than LIS. The results of the meta-analysis indicted that LIS was superior in terms of recurrence and healing rates (18, 19). Another study found 93.1% for post-LIS healing rate and 62.6% for BoNT injection (1). The risk of permanent damage to anal sphincter has called for the application of alternative treatments such as BoNT injection. The aim of this study is to compare the effectiveness of BoNT which is a less invasive procedure and redo-LIS for treatment of post-LIS recurrent anal fissure.

## Methods

The study involves 19 patients who received LIS treatment and then redo-LIS or BoNT injection due to recurrence in the follow-up. Observing the criteria of Helsinki Declaration, approval was obtained from the ethics board. Files of 118 patients who had received LIS and BoNT injection for chronic anal fissure were reviewed. Patients who had inflammatory bowel disease, prior anorectal surgery for non-fissure reasons, underlying hemorrhoidal condition and/or fistula, presence or suspicion of malignity were excluded from the study. The 19 patients who were included in the study in accordance with the methodology had developed post-LIS recurrent anal fissure and received redo-LIS or BoNT injection. These patients were assessed through standardized clinical forms, their medical history and files were reviewed in detail, and anorectal examinations were conducted.

Using standardized forms, patients' age, sex and complaints (pain, bleeding, continence and recurrence) were recorded. **Study groups had patients who chose redo-LIS or BoNT**. Two groups were formed as Group I including patients who received redo-LIS, and Group II including those who received BoNT application.

The redo-LIS group (Group I) included patients who had received LIS for chronic anal fissure, then were given redo-LIS due to pain or recurrence the 3-month post-surgery follow-up period.

Redo-LIS was performed as internal sphincterotomy by incision through LIS scar contra-lateral through fissure apex under sedation and local anaesthesia, in the prone jack-knife position.

**The BoNT group (Group II) included patients who had received LIS for chronic anal fissure, then developed recurrence or failed to heal, but did not want repeat surgical treatment and had incontinence anxiety**. BoNT was applied, under sedation, by injection into the internal sphincter from two laterals in the form of two insulin injectors each containing 0.5 ml of solution containing 100 IU Botulinum Toxin type A diluted with 1 ml of physiological saline solution.

After the procedure, patients were instructed, for 2 weeks, to have high-fibre diet and warm sitz-bath three times a day.

Both groups were called in for control in the 1st week, 1st month and 3rd month, and examination findings, pain and continence scores were recorded through standardized forms. The criteria for fissure healing were adopted as fissure epithelization and complete disappearance of pain during and after defecation. The status of continence was assessed using the Cleveland Clinical Incontinence Score (CCIS) system (20). The data were statistically analysed using Statistical Package for Social Sciences (SPSS 22). **The results were accepted statistically significant with *p* < 0.01 within the confidence interval of 95%.**

## Results

The 19 patients included in the study had previously received LIS for anal fissure. Of the 19 patients, 11 were male (57.89%) and 8 were female (42.10%) with average age of 42.37 ± 5.96. Table 1 presents the sex and average age of patients in Group I and Group II. There was no statistically significant difference by age or sex between groups (*p* = 0.89, *p* = 0.13 respectively). Of the patients, 11 (57.89%) were performed redo-LIS, with 8 being male (73%), and 3 being female (27%). Of the 8 patients who were applied BoNT, 3 were male (38%) and 5 were female (62%).

**Table 1:** Study group status by age and sex.

	Group I (Redo-LIS)	Group II (BoNT)	*P* value
Male	8 (avg. age 46, 5)	3 (avg. age 33.6)	0.13^a^
			0.89^b^
Female	3 (avg. age 51.3)	5 (avg. age 43, 2)	0.13^a^
			0.89^b^

^a^ Age.

^b^ Sex.

Pain was present in all patients prior to intervention. Following the intervention there was statistically significant difference **(*p* = 0.008)** between groups by pain (Table 2).

**Table 2:** Study group status by pain and incontinence.

	Group I (redo-LIS)	Group II (BoNT)	*P* value
Preop CCIS	0, 36	0, 25	0.87
Postop 3rd month CCIS	0, 12	0, 13	0.83
Preop VAS	7, 64	7, 62	0.13
Postop 3rd month VAS	0.09	7.25	0.008*

CCIS, cleveland clinic incontinence score; VAS, visual analogue score.

Comparisons were peformed by the Mann Whitney *U*-test.

*p < 0.01 compared with the pre-operative VAS score.

Of redo-LIS recipients, one had minor bleeding at the incision location that did not require intervention in the post-surgery period. Of BoNT recipients, one had ecchymosis at the injection area, later receded spontaneously.

For incontinence, one of the patients in Group I had mild gas incontinence (CCSI = 3), and no patient in Group II had incontinence. The 3rd-month follow-up of patients resulted in complete disappearance of incontinence complaint in Group I as well. There was no statistically significant difference between groups by pre- or post-surgery incontinence (by CCIS) of both groups (*p* = 0.87; *p* = 0.83 respectively). Table 2 presents the status of patients by incontinence and pain**. In group I healing rate was 100%, while in group 2 (BoNT group) two of eight patients recurrence were assessed.**

## Discussion

Anal fissure has a vicious cycle characterised by internal sphincter spasm, pain and bleeding during defecation (21). The fundamental objective of treating anal fissure is to reduce internal sphincter pressure to normal levels. To treat acute anal fissure, warm-sitz baths and cremes that reduce internal sphincter pressure are used (3).

When the condition becomes chronic, LIS or BoNT injection may be applied. LIS is the gold standard for treating chronic anal fissure**. BoNT injection into internal sphincter is used due to the risk of incontinence though low, for cases** where the patient has some clinical risks for surgery (undergoing chemotherapy, high cardiac risk, requirement to use blood diluents etc.) or **the patient avoids surgical treatment** (1, 6, 11, 12).

Studies reported recurrence rates following LIS treatment of chronic anal fissure as 1.3% to 25% (21, 22). Post-LIS recurrence could go down to 0.3% if applied in clinics experienced in proctology (23). This may be associated with the more effective and complete performance of the LIS procedure.

Based on our clinical experience, one of the reasons for failed surgical treatments of chronic anal fissure is the selected method of anaesthesia. Muscle relaxing drugs administered to the patient for the LIS under general anaesthesia or the spinal anaesthesia affect internal sphincter causing relaxation and consequently difficulty in surgical dissection. For such patients, any difficulty in dissecting internal sphincter may result in inadequate sphincterotomy.

Our clinical experience also leads us to think that the performance of LIS procedure under sedation and local anaesthesia improves the visibility of internal sphincter, thus allow better dissection, resulting in a more successful LIS or avoiding an unsuccessful one. LIS patients that receive LIS by this method do not have stay in the hospital following LIS.

For cases where recurrence have developed, treatment approaches are still discussed. The top reason for recurrence is inadequate sphincterotomy.

In case of anal fissure that recurs or continues following an inadequate/inappropriate LIS, surgeons or patients mostly avoid redo-LIS. The reason is that a redo-LIS will likely exacerbate the damage to anal sphincter which in turn creates a heightened risk of anal incontinence. This may lead to preferring BoNT injection for recurrent fissures (1).

We compared the success rates of treatment of patients by redo-LIS or BoNT injection due to developing recurrence after having received LIS for chronic anal fissure. Our study demonstrates that, at the clinics experienced in proctology, the most dreaded risk of incontinence following redo-LIS is at acceptable levels at the early phase and returns to normal at the end of 3rd month. Its rates were reported in the literature as 0.4% to 35% (23, 24).

As for the assessment of pain following redo-LIS and BoNT procedures, the BoNT group was observed to have continued pain following defecation which was statistically significant (*p* = 0.008). There is no study in the literature comparing BoNT and redo-LIS for treating recurrent anal fissures. One sole study reports 4% for the recurrence rate following redo-LIS for treating recurrent anal fissure (21).

In our study, BoNT application was found to be less successful for the patient group who had previously received surgery. We conjecture that the reason is the internal sphincter fibrosis related to the previous LIS which reduces the effectiveness of BoNT. BoNT application for treating chronic anal fissure may be less successful for those who previously had anorectal surgery than those not.

Redo-LIS is a reliable, successful method for patients who developed recurrence or did not heal following LIS. The performance of the redo-LIS by incision through previous scar's contra-lateral may facilitate dissection and contribute to adequate performance of sphincterotomy.

## Conclusion

This study demonstrates that redo lateral internal sphincterotomy (LIS) is a reliable method for patients who received LIS but developed recurrent chronic anal fissure, and achieves successful results in terms of recurrence and relief of pain. BoNT application is less successful for patients group who previously received surgery and developed recurrence than patients who did not receive surgical treatment. The likely reason is the internal sphincter fibrosis related to the previous LIS which reduces the effectiveness of BoNT.

## Study limitations

This study was limited because it was a single-armed, retrospective analysis of prospectively designed data.

## Data Availability

The original contributions presented in the study are included in the article/Supplementary Material, further inquiries can be directed to the corresponding author/s.
